# Oncogenic Landscape of Somatic Mutations Perturbing Pan-Cancer lncRNA-ceRNA Regulation

**DOI:** 10.3389/fcell.2021.658346

**Published:** 2021-05-17

**Authors:** Yuanfu Zhang, Peng Han, Qiuyan Guo, Yangyang Hao, Yue Qi, Mengyu Xin, Yafang Zhang, Binbin Cui, Peng Wang

**Affiliations:** ^1^College of Bioinformatics Science and Technology, Harbin Medical University, Harbin, China; ^2^Department of Colorectal Surgery, Harbin Medical University Cancer Hospital, Harbin, China; ^3^Heilongjiang Cancer Research Institute, Harbin, China; ^4^Department of Gynecology, The First Affiliated Hospital of Harbin Medical University, Harbin, China; ^5^Department of Anatomy, Harbin Medical University, Harbin, China

**Keywords:** ceRNA, somatic mutation, prognosis, functional analysis, oncogenic pathway

## Abstract

Competing endogenous RNAs (ceRNA) are transcripts that communicate with and co-regulate each other by competing for the binding of shared microRNAs (miRNAs). Long non-coding RNAs (lncRNAs) as a type of ceRNA constitute a competitive regulatory network determined by miRNA response elements (MREs). Mutations in lncRNA MREs destabilize their original regulatory pathways. Study of the effects of lncRNA somatic mutations on ceRNA mechanisms can clarify tumor mechanisms and contribute to the development of precision medicine. Here, we used somatic mutation profiles collected from TCGA to characterize the role of lncRNA somatic mutations in the ceRNA regulatory network in 33 cancers. The 31,560 mutation sites identified by TargetScan and miRanda affected the balance of 70,811 ceRNA regulatory pathways. Putative mutations were categorized as high or low based on mutation frequencies. Multivariate multiple regression revealed a significant effect of 162 high-frequency mutations in six cancer types on the expression levels of target mRNAs (ceMs) through the ceRNA mechanism. Low-frequency mutations in multiple cancers perturbing 1624 ceM have been verified by Student’s *t*-test, indicating a significant mechanism of changes in the expression level of oncogenic genes. Oncogenic signaling pathway studies involving ceMs indicated functional heterogeneity of multiple cancers. Furthermore, we identified that lncRNA, perturbing ceMs associated with patient survival, have potential as biomarkers. Our collective findings revealed individual differences in somatic mutations perturbing ceM expression and impacting tumor heterogeneity.

## Introduction

In recent years, novel, post-transcriptional regulatory mechanisms of competing endogenous RNAs (ceRNAs) have been revealed. RNA molecules, including messenger RNAs (mRNAs), long noncoding RNAs (lncRNAs), circular RNAs, and pseudogene transcripts, can function as competing endogenous RNAs (ceRNAs) to indirectly regulate the expression of relevant target genes by competing with each other for microRNAs (miRNAs) (Salmena et al., 2011). These ceRNAs harbor miRNA response elements (MREs) that bind to miRNA through complementary sequences and can induce degradation or inhibition of the expression of target genes. In addition, the combination of miRNAs and target genes was a complex network; one miRNA can regulate multiple genes and one gene can be regulated by multiple miRNAs.

LncRNAs were once regarded as byproducts of gene transcription (Quinn and Chang, 2016). However, they are crucial in post-transcriptional regulation through the ceRNA mechanism (Chen et al., 2019) and are dramatic factors that contribute to biological growth and development, aging, diseases, and multiple cancers (Rinn and Chang, 2012; Schmitt and Chang, 2016). For example, MALAT1, which is highly expressed in most cancers, regulates the cell cycle (Tripathi et al., 2013), and PCA3 is an important molecular marker in the early stage of cancer (Lemos et al., 2019). Somatic mutations, which occur in cells other than germ cells and are not inherited, are the substantial cause of most tumors (Jia and Zhao, 2017). Mutation in an miRNA response elements (MRE) of lncRNA can weaken, enhance, or prevent binding to the original miRNA, resulting in an imbalance in the ceRNA regulatory network and altered expression of the relevant target genes (Thomas et al., 2011; Thomson and Dinger, 2016).

The development of sequencing technologies has enabled the identification of somatic mutations associated with tumors (Martincorena and Campbell, 2015). Genetic variations affecting miRNA gene expression have been described (Civelek et al., 2013; Siddle et al., 2014), as has the expression of coding genes whose 3′ untranslated regions are targeted by miRNAs (Gamazon et al., 2012; Lu and Clark, 2012). Genetic polymorphisms affecting the regulation of human ceRNAs have been reported (Li M. J. et al., 2017). However, few studies have explored the effects of somatic mutations on ceRNA mechanisms.

Here, we used mutation and RNA-seq profiles from The Cancer Genome Atlas (TCGA) (Tomczak et al., 2015) database to conduct a systematic investigation concerning the effects of lncRNA mutations on the expression of target mRNAs via the ceRNA mechanism in pan-carcinoma. We also studied the impact of significant mutations on oncogenic mechanisms and patient survival.

## Materials and Methods

### Data Collection

Information concerning RNA-seq and somatic mutation profiles of 33 cancers were obtained from The Cancer Genome Atlas (Tomczak et al., 2015) (TCGA)^1^ database. The GRCh38 v29 version of the human genome annotation data from GENCODE (Harrow et al., 2012)^2^, including the position and sequence information of lncRNAs, was used to annotate somatic mutation profiles. Sequences of miRNA and annotation information were obtained from the miRBase (Kozomara and Griffiths-Jones, 2014)^3^ database. Interaction data of miRNA and target genes (mRNA) that were validated using established experimental methods including the luciferase reporter assay, PCR, and western blotting were collected from miRTarBase (Chou et al., 2018) V8.0^4^. Hallmark (Hanahan and Weinberg, 2011) gene sets were collected from the Molecular Signatures Database (MSigDB Liberzon et al., 2011)^5^.

### Construction of Somatic Mutation-miRNA-lncRNA (ceL)-mRNA (ceM) Unit

Among the numerous somatic mutations in the pan-cancer genome, lncRNA mutations was the focus of this study. We, respectively, define the lncRNA and mRNA involved in the ceRNA regulatory mechanism as ceL and ceM. Sequences approximately 7 nucleotide (nt) upstream and downstream of the lncRNA somatic mutation sites were extracted using the lncRNA annotation from GENCODE, which will be used to construct mutation and control sequences. Considering that the TargetScan (Friedman et al., 2009) software does not recognize short sequences, it was necessary to extract longer upstream nucleotide sequences (14 nt) to offset this impact. TargetScan and miRanda (Betel et al., 2008) (e.g., the miRanda algorithm) are miRNA target gene prediction tools, and therefore were used to predict the miRNA-target relationships of control sequences with strict thresholds of score > 160 and energy < −20 for miRanda and context score < −0.4 for TargetScan. We defined the lncRNA and mRNA involved in the imbalance of ceRNA regulatory mechanism as ceL and ceM, respectively. We selected “mutation-miRNA-lncRNA (ceL)” units with varying binding affinities between the mutation and control sequences, and regarded loss, down, gain, and up as the four conditions of altered lncRNA and miRNA binding affinity (Li M. J. et al., 2017). We further searched for candidate mRNAs (ceMs) controlled by the same miRNA from the miRNA-target gene data of miRTarBase as the last element to construct the somatic mutation-miRNA-ceL-ceM (SMILM) unit. In this context, “putative mutations” are defined as mutations effecting original ceRNA regulation mechanism. This definition has been used in a previous study of genetic associations with ceRNA regulation in the human genome (Li et al., 2020).

### Classification and Definition of Mutations

Somatic mutations do not occur frequently. We defined a site at which at least two samples displayed a mutation as a high-frequency (HF) mutation site. The remaining mutations were defined as low-frequency (LF) mutation sites. The altered binding affinity of lncRNA and miRNA binding was divided into four states (gain, up, loss, and down). Gain, up, loss, and down were scored as +1, +0.5, −1, and −0.5, respectively. The functional score of each mutation was calculated by summing the states of all mutated miRNAs associated with it. For LF mutations, we focused on the mRNAs (ceMs) affected by somatic mutations through ceRNA regulatory mechanisms. The possible expression tendency of the mRNA was defined as “up” means that the sum of the mutation scores that regulate this mRNA is greater than zero, “down” means the opposite of “up,” and “none” means that the sum of the mutation scores that regulate this mRNA is equal to zero.

### Multivariate Multiple Regression Analyses

Different experimental tools were used for identification of SMILM units mediated by HF mutations (HF-SMILM) and LF mutations (LF-SMILM). In the HF-SMILM unit, multivariate multiple regression models were used to validate whether the expression level normalized by Fragments PerKilobase Million (FPKM) of ceL and ceM conformed to target prediction results (Valente et al., 2014). The fold-change values were used to evaluate the extent of expression changes between two groups of samples. For each SMILM unit, we considered the expression levels of lncRNA (El) and mRNA (Em) as two independent response variables. As a predictor, the genotype (Gt) of an individual was used as the driving variable. Synergistic factors such as the residual expression of miRNAs might also affect the response variables. At the same time, we assumed that the error vector ε = (ε_1_, ε_2_)′ followed a multivariate Gaussian distribution with an expected value of zero and an unknown covariance matrix. The multivariate multiple regression model constructed for the SMILM unit is:

(1) (El,Em)=Gt+MIr+ε

We used this equation to validate all the SMILM units. We defined *ηl* and *ηm* as the regression coefficients of the driving variable Gt. The influence of Gt changes on the expression of ceL and ceM was quantified using the regression coefficients *ηl* and *ηm*, and the statistical significance of the model was obtained. Since ceL and ceM present a competitive relationship in the ceRNA mechanism, we required that ceL expression changes with genotype and ceM expression changes with genotype followed opposite tendencies (*ηl* × *ηm* < 0, *p*−value < 0.05) (Li M. J. et al., 2017).

LF-SMILM unit data were split based on the characterization by ceM, obtaining the somatic mutations, lncRNA, miRNA, and samples corresponding to each ceM. We divided cancer samples into mutated and non-mutated samples according to whether the sample had mutations that affected the expression of specific mRNA (ceM) through the ceRNA mechanism. Student’s *t*-test was used to compare the ceM expression changes in the two categories of sample. The ceMs with *p*-value < 0.05 were retained due to significant changes in their expression affected by putative mutations.

### Construction of ceRNA Regulatory Network

In the SMILM unit validated by multivariate multiple regression models, the mutated lncRNA (ceL), miRNA, and target gene mRNA (ceM) constitute a two-level regulatory relationship. Therefore, we used Cytoscape (Shannon et al., 2003) to visualize this regulatory relationship in significant SMILM units [mutations-miRNA-lncRNA (ceL)-mRNA (ceM)] (Long et al., 2019).

### Functional Enrichment Analysis Connecting Oncogenic Signaling Pathways

We obtained ceMs whose expression were significantly affected by somatic mutations through the ceRNA mechanism. To assess the role of these ceMs in various cancers, we used the compareCluster function in the R package clusterProfiler to perform functional analyses on multiple pan-cancer gene sets, using threshold pvalueCutoff = 0.05. Seventeen oncogenic signaling pathways (Malumbres and Barbacid, 2003; Reya and Clevers, 2005; Wade et al., 2013; Moradi-Marjaneh et al., 2018; Ayuk and Abrahamse, 2019; Soleimani et al., 2019, 2020) were collected from articles published between 2008 and 2019. The overlapping signal path was filtered out based on enrichment results.

### Survival Analysis

We used the Cox proportional hazards model (Fisher and Lin, 1999) to estimate whether the expression of ceMs regulated by lncRNA mutations according to the ceRNA mechanism was related to patient survival. Hazard ratios (HRs) < 1 and *p* < 0.05 indicated significant relationships between ceM and reduced risk of death. An HR > 1 indicated the converse. Based on the predicted results, each sample was categorized as one of four types: including “None” means no mutation disrupts the expression of the target gene, “Up-regulated” means the presence of mutations that cause only upregulation of target gene expression, “Down-regulated” means the presence of mutations that cause only downregulation of target gene expression, and “Unknown” means that both mutations resulting in up- and down-regulation of the expression of the target gene are present. The R package for survival was used to create survival curves (Rich et al., 2010). The fitted results were visualized using a ggsurvplot. A *p*-value < 0.05 was considered to represent a significant difference in survival.

## Results

### Global Mutation Map Reveals Heterogeneity of Different Tumors

**FIGURE 1:**
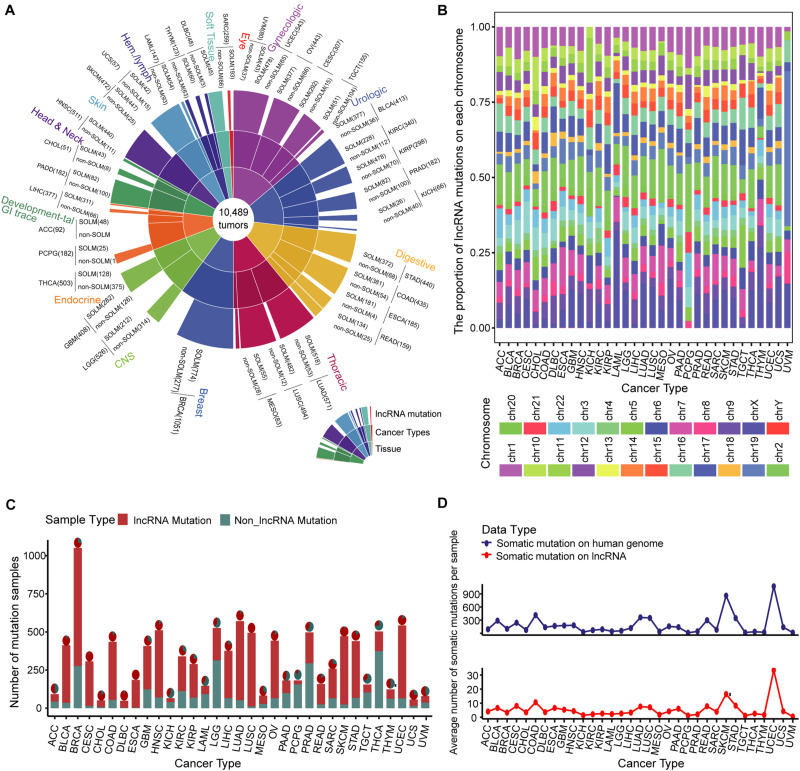
Landscape of somatic mutations across 33 cancer types. **(A)** Global map of lncRNA mutations in different tissues and cancers. **(B)** Bar plot of the proportion of lncRNA mutations on each chromosome in multiple cancer types. Twenty-two pairs of homologous chromosomes and two sex chromosomes are marked with distinct colors. **(C)** Samples with lncRNA somatic mutations are marked in red in 33 cancer type samples. The pie chart illustrates the proportion of lncRNA mutations in all somatic mutations. **(D)** Average numbers of somatic mutations per sample on the entire genome and lncRNA are presented by line chart across 33 cancer types.

We evaluated samples from the TCGA database collection for which somatic mutation data were available, producing a global map of somatic mutation sample distribution. The map contained 7604 samples with lncRNA mutations in 10,489 samples from 33 cancers (Figure 1A and Supplementary Table 1). We examined the distribution of mutations on chromosomes. The lncRNA mutations in multiple cancer types aggregated differently on chromosomes, especially in kidney renal papillary cell carcinoma (KIPR), acute myeloid leukemia (LAML), pheochromocytoma and paraganglioma (PCPG), thymoma (THYM), and uveal melanoma (UVM), compared to those in other tumors (Figure 1B). These findings indicate that the distribution specificity of lncRNA mutations on chromosomes may be the underlying cause of cancer functional heterogeneity. Of all renal cell carcinoma subtypes, the KIPR subtype of kidney cancer has different molecular characteristics and poor survival (Cancer Genome Atlas Research Network, Linehan et al., 2016; Ricketts et al., 2018). Lung adenocarcinoma (LUAD) and lung squamous cell carcinoma (LUSC) lung cancer subtypes displayed similar mutation distribution profiles on chromosomes, suggesting that cancers in the same tissue site have a similar distribution of mutations on chromosomes. Breast invasive carcinoma (BRCA), kidney chromophobe (KICH), kidney renal clear cell carcinoma (KIRC), and thyroid carcinoma (THCA) displayed large sample sizes but relatively few lncRNA mutations, suggesting that mutations in lncRNAs have a strong distribution preference among various cancers (Figures 1C,D). LncRNA mutations had low frequencies in the range of 1 to 10%, suggesting that the rates of lncRNA mutations vary among cancer types (Supplementary Figure 1). These findings were consistent with previous studies showing that mutation frequency fluctuates significantly in pan-cancer, and that the mutation rate of some cancers is greatly increased due to missing repair pathways or chromosome integrity checkpoints (Martincorena and Campbell, 2015). We also assessed numbers of mutations per lncRNA in cancer types. A set of lncRNAs with a high mutation frequency was evident for multiple cancers (Supplementary Figure 2). The lncRNAs XIST, TTN-AS1, STRA6LP, and TSIX had high numbers of mutations in most cancers, which play an important role in the oncogenic mechanism. The lncRNA XIST can regulate X chromosome silent transcription and act as an miRNA sponge upregulating SOD2 to inhibit the development of non-small cell lung cancer (Chen et al., 2016; Liu et al., 2019). TTN-AS1 is an miRNA sponge that regulates cancer development through a ceRNA mechanism (Wang Y. et al., 2020).

### Significant Mechanism of lncRNA Mutation Perturbing ceRNA Regulation

**FIGURE 2:**
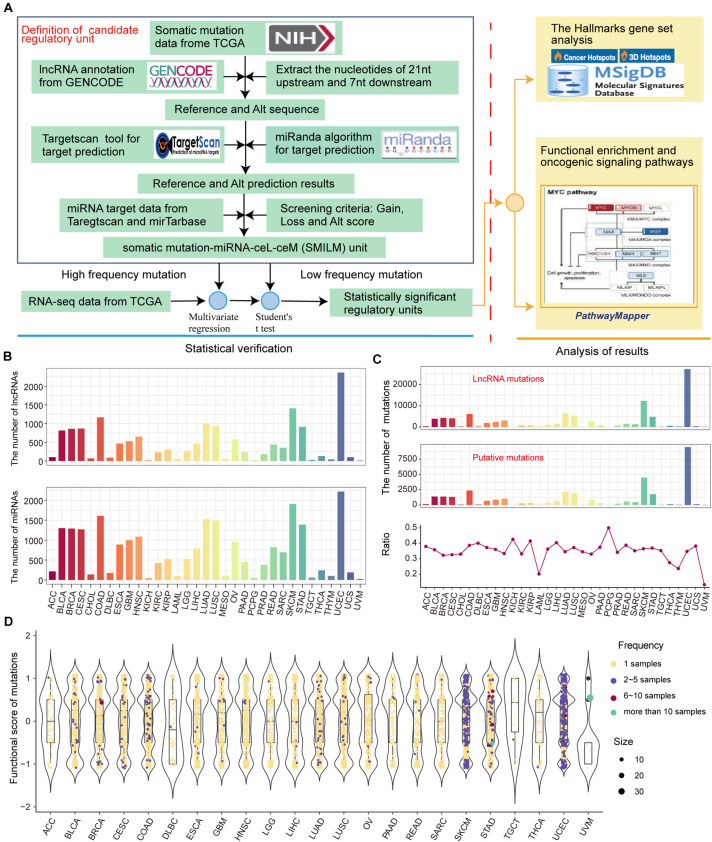
Predicting somatic mutations on lncRNAs that potentially impact the ceRNA mechanism. **(A)** Workflow for SMILM unit construction, unit verification, and functional pathway analysis. **(B)** Numbers of lncRNAs and miRNAs affected by somatic mutations in different cancers. **(C)** Numbers of putative somatic mutations in SMILM units across 33 cancers is shown in a line chart. Proportions of putative mutations in each cancer type as a percentage of the original somatic mutations on lncRNA are shown by bar plot. **(D)** Functional scores of putative mutations in each cancer. Blue, red, green, and purple denote numbers of samples in which a particular mutation occurs.

We developed a pipeline to assess the effect of somatic mutations perturbing lncRNA-ceRNA regulation in pan-cancer (Figure 2A). To examine the influence of lncRNA mutations on miRNA binding sites according to TargetScan (Friedman et al., 2009) and miRanda (Betel et al., 2008), we used pan-cancer mutation profiles from TCGA. In total, we identified 31,560 putative somatic mutation sites for 33 cancer types in 3,124 putative miRNA target genes (putative lncRNAs). These mutated lncRNAs showed different binding affinities to 2437 miRNAs compared to wild type sequences across 33 cancers (Figure 2B). Considering that the larger numbers of putative mutations in several cancers are due to larger numbers of initial mutations, the proportions of putative mutations compared with the original lncRNA mutations were examined, which reflected the contribution of the mutations-miRNA-ceRNA mechanism in the carcinogenic process across the 33 cancers. PCPG, which had lower number of mutations and average number of mutations per sample, had the highest percentage of putative lncRNA mutations to original somatic mutations on lncRNA (Figure 2C), suggesting that the contribution of the mutations-miRNA-ceRNA mechanism in the oncogenic process is not determined simply by numbers of mutations. Next, for each putative lncRNA (ceL), we found other experimentally verified mRNAs (ceM) targeted by the same miRNA and established a minimal miRNA-ceRNA regulation unit, which we termed the somatic mutation-miRNA-ceL-ceM (SMILM) unit. Taken together, these results reveal significant mechanisms by which mutations perturb gene expression.

The frequency of somatic mutations is lower compared with genetic variations, and an appropriate number of mutation and control samples to explain the relationship between ceL and the corresponding ceM expression in SMILM units is not available (Li M. J. et al., 2017). Therefore, we defined mutations in at least two samples of the same cancer as high-frequency mutations (HF mutations, *n* = 831), and the rest as low-frequency mutations (LF mutations, *n* = 32,823) (Figure 2D). For these two categories of mutations, we separately applied multiple regression and Student’s *t*-test to jointly model the contribution of mutations on ceRNA expression variation (see section Materials and Methods).

### Statistical Identification Portrays ceRNA Expression Fluctuation Landscape

#### ceRNA Expression Variation Driven by HF-Mutation

**FIGURE 3:**
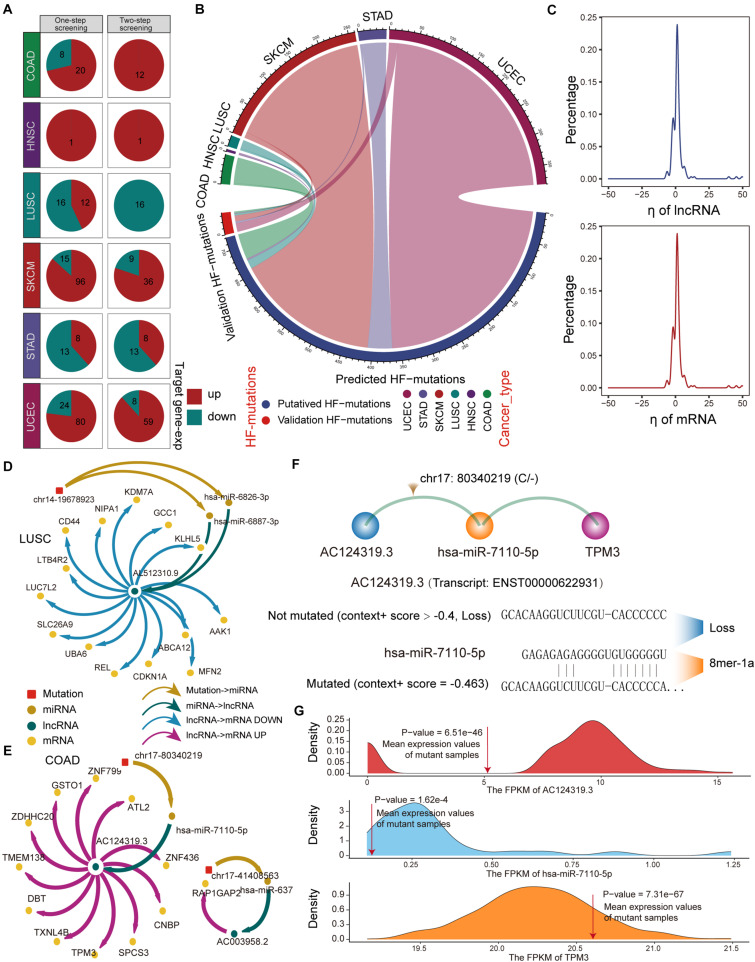
Regression model to identify HF-SMILM units. **(A)** HF-SMILM units that passed one-step and two-step screening in six cancer types. Directions of change in the expression of ceM in the HF-SMILM unit affected by the mutation are marked by blue and red. **(B)** Circle diagram of proportions of putative mutations in these six cancers before and after the regression test. **(C)** Distribution of regression coefficients of ceL and ceM in the multiple regression analysis of the HF-SMILM units. **(D)** Regulatory network of HF-SMILM units that passed regression test in LUSC. Unique identifiers used by nodes and interactions in the network. **(E)** Same as in **(D)** but for HF-SMILM units that passed regression test in COAD. **(F)** Binding affinity changes of AC124319.3 and hsa-miR-7110-5p before and after the chr17: 80340219 (C/–) mutation in AC124319.3. **(G)** The density curve reflects the distribution of genes expression for COAD and the average expression level of the genes in the mutant sample was marked with a red line. The one-sample *t*-test was used to calculate statistical significance.

Next, we used a regression model to examine the effect of HF mutations on ceRNA expression levels. We found that only six cancer types, including colon adenocarcinoma (COAD), head and neck squamous cell carcinoma (HNSC), LUSC, skin cutaneous melanoma (SKCM), STAD, and uterine corpus endometrial carcinoma (UCEC), had putative HF mutations that passed the regression test and identified 293 ceL and ceM genes whose expression levels were significantly correlated with genotypic changes (Figure 3A). Several factors affecting the effectiveness of ceRNAs have been reported, including the expression levels of miRNAs and ceRNAs, as well as binding affinity to miRNA target sites (Ebert and Sharp, 2010; Mukherji et al., 2011; Salmena et al., 2011). Since miRNA expression variation has been considered in the regression model, we focused on ceRNA-centric factors. We further required a consistent direction between the regression coefficient η*l* and the changes in the functional prediction score from TargetScan and miRanda. Accordingly, we redefined 162 SMILIM units, in which ceL and ceM expression variations displayed opposite and consistent orientations with the target prediction results (Figure 3A). Among 742 putative transition and transversion mutations, 17 mutations were identified to disturb the ceRNA regulation. In addition, three of 59 putative indel mutations were found to disturb the ceRNA regulation. Compared to the original HF mutations, the verified somatic mutations were drastically reduced (Figure 3B). It is likely that ceM expression changes rely not only on a minimal SMILM unit, but also on the interaction of the ceRNA network and other regulatory factors, such as transcription factors (TFs) and DNA methylation.

The majority of η*l* and η*m* were concentrated between −10 and 10 in the 162 significant SMILM units, suggesting an important effect of these somatic mutations on ceM expression and ceRNA regulation compared with genetic variation (Li M. J. et al., 2017; Figure 3C). The mutation-ceRNA regulatory relationship was a complex network, where a single mutation affected the affinity to bind multiple miRNAs and thus disturbed the expression of multiple mRNAs (Figures 3D,E and Supplementary Figure 3). For example, an indel (chr17: 80340219C) of COAD enhanced the binding affinity of hsa-miR-7110-5p in lncRNA AC124319.3 (ENSG00000280248; *p-*value = 3.57E-3, η*l* = -8.03), which competed with TPM3 (*p-*value = 6.43E-5, η*m* = 0.79; Figures 3E,F). Further, one-sample *t*-test confirmed that the expression levels of AC124319.3, hsa-miR-7110-5p, and TPM3 in non-mutated samples were statistically different from the average expression of mutant samples (Figure 3G), suggesting that the expression level of miRNA, as a key link with the ceRNA regulatory pathway, is an important factor in identifying the imbalance in the ceRNA mechanism. Taken together, these results suggest that the presence of somatic mutations in lncRNAs could affects the expression of target genes through a ceRNA regulatory pathway.

#### Individual Differences in ceM Expression Variation Produced by LF-Mutation

**FIGURE 4:**
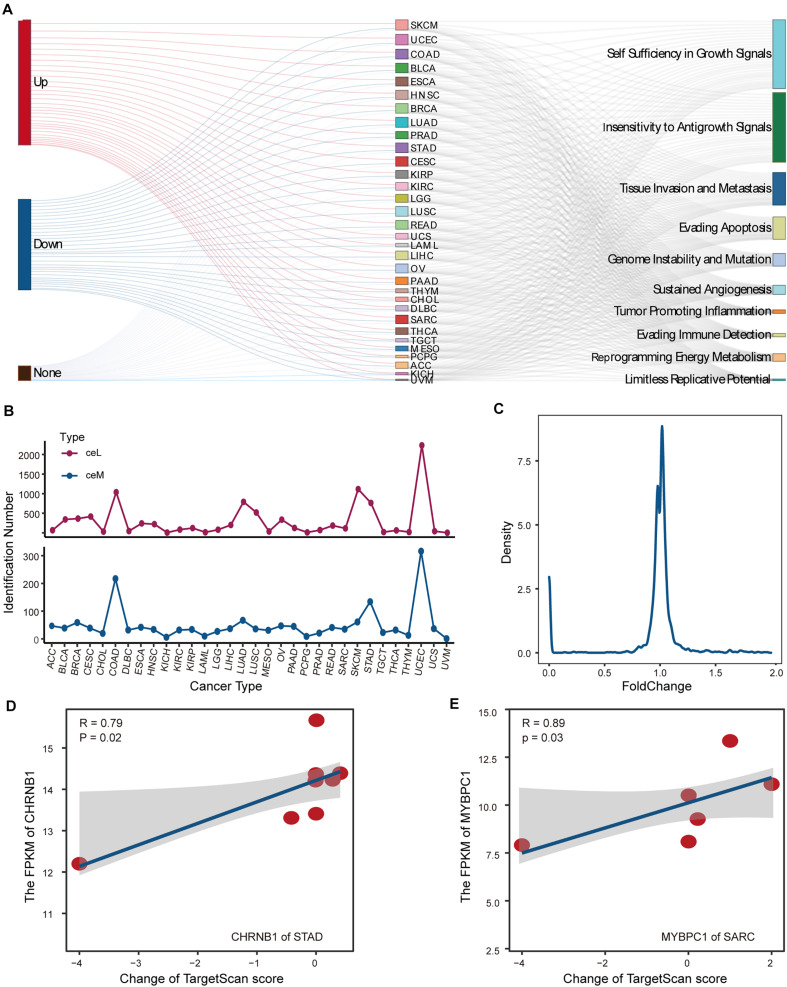
Analysis and detection of LF-mutations. **(A)** Sankey diagram demonstrating the possible effects of putative LF-mutations on the corresponding ceM expression and the contribution of these ceMs to the 10 classical hallmark gene sets. **(B)** Numbers of ceM and ceL that passed the statistical test in pan-cancer. **(C)** Line plot illustrating the density distribution of fold change of all ceM that passed the statistical test in pan-cancer. **(D)** Relationship between expression of CHRNB1 in STAD and the corresponding lncRNA-miRNA target prediction scores shown by scatter plots with plotting of fitted curves. **(E)** Same as in **(D)** but for the expression of MYBPC1 in SRAC.

For SMILM units disturbed by LF mutations, we focused on the target ceMs determined by the operability of the experiment and the important role of protein-coding genes in physiological function. To assess the carcinogenic function of LF-SMILM units, we extracted target ceMs involved in cancer hallmarks, which are important biological processes in cancer development (Agarwal et al., 2015). We divided the expression changes of the target ceM into up, down, and none (Figure 4A, see Supplementary Methods). Our data suggest that lncRNA mutations amplify and depress the expression of protein-coding genes (ceMs) through the ceRNA mechanism in multiple cancers to comprehensively impact the carcinogenic process of the hallmarks.

We statistically verified the expression variation of hallmark ceMs affected by LF mutations through the ceRNA mechanism. We found that the expression levels of 1624 ceMs occurring in 32 cancer types were significantly different between the corresponding mutant and control samples and were regulated by 2849 ceL. Cancers with a high number of samples or high lncRNA mutations displayed a small number of identified ceMs (Figure 4B), suggesting that the contribution of mutation-miRNA-ceRNA mechanism is heterogeneous in pan-cancer. Fold change as a measure of change in ceMs expression was found to be clustered between 0.8 and 1.2 (Figure 4C), suggesting that simple statistical metrics mask individualized differences in the expression of mutant interference ceMs. We also found that variation in the expression of target ceM was highly correlated with the change in target prediction score in STAD and Sarcoma (SARC) (Figures 4D,E). This evidence suggests that a ceM could be affected by multiple SMILM units, which have different regulatory effects on the expression of ceM determined by individual differences in putative mutations. Together, these data indicate that individual mutation differences are an important cause of fluctuations in the expression of ceMs.

#### ceM Oncogenic Pathways Reveal Pan-Cancer Functional Heterogeneities

**FIGURE 5:**
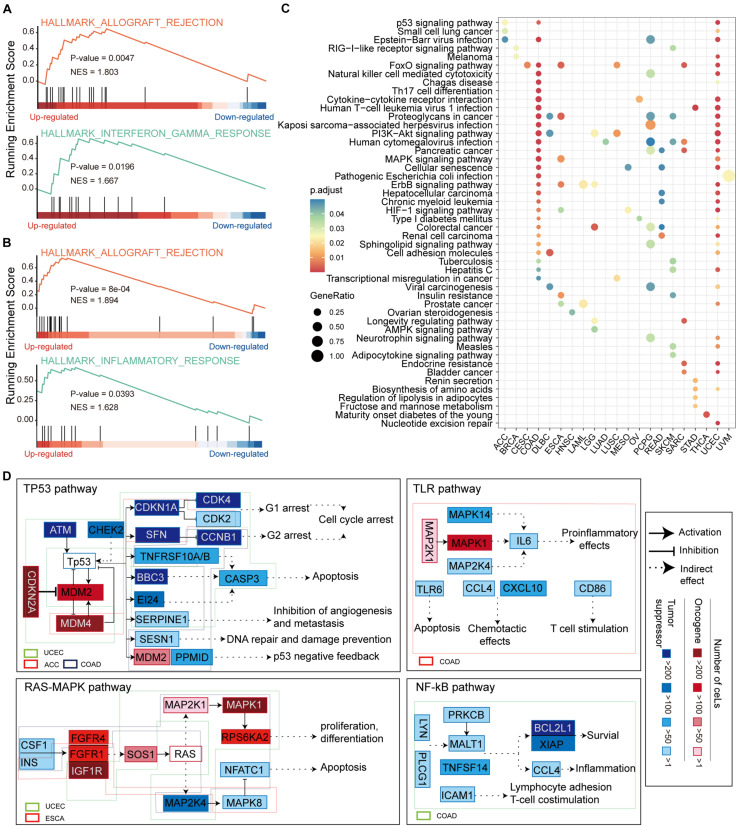
Functional enrichment analysis of ceMs. **(A)** Enrichment of allograft rejection and inflammation response pathways on ceMs for COAD. **(B)** Same as in **(A)** but for UCEC. **(C)** Dot plot illustrating the top five pathways from functional enrichment results of ceMs for pan-cancer. **(D)** Signaling pathways affected by ceM mapped to illustrate the function mechanism of ceM in pan-cancer. Oncogenic and suppressor genes are shown in red and blue, respectively. Use of three categories to represent the relationships between two genes: activation, inhibition, and indirect effect.

CeMs confirmed to be affected by LF mutations in pan-cancer were collected for gene set enrichment analysis (GSEA) (Subramanian et al., 2005) weighing by fold change of ceM expression. We found significantly enriched hallmark gene sets in ceM genes only in COAD and UCEC. Allograft rejection and inflammation response pathways were enriched in ceMs with upregulated expression in both COAD and UCEC (Figures 5A,B), revealing a high similarity in the effects of lncRNA mutations through ceRNA mechanisms in COAD and UCEC. Further, we integrated all ceMs perturbed by HF and LF mutations to analyze the effect of mutation-miRNA-ceRNA mechanism on cellular functions in pan-cancer by KEGG functional enrichment analysis. We found only 21 cancers with significantly enriched functional pathways, primarily involved in energy metabolism, cell metastasis, apoptosis, and functions related to cancer complications (Figure 5C and Supplementary Figure 4), indicating that the mutations-miRNA-ceRNA mechanism is widely involved in the development of cancers.

We compiled and reviewed 17 oncogenic signaling pathways verified in articles published between 2008 and 2019 (Supplementary Table 2). We only identified eight oncogenic signaling pathways in the KEGG functional enrichment results of eight cancer types (Supplementary Figure 5). These include the p53 signaling pathway (Wade et al., 2013), phosphoinositide 3-kinase (PI3K)-AKT signaling pathway, mitogen-activated protein kinase (MAPK) signaling pathway (Soleimani et al., 2019), Ras signaling pathway (Malumbres and Barbacid, 2003), Toll-like receptor (TLR) signaling pathway (Moradi-Marjaneh et al., 2018), mammalian target of rapamycin (mTOR) signaling pathway (Ayuk and Abrahamse, 2019), Wnt signaling pathway (Reya and Clevers, 2005) and NF-kappa B (NF-kB) signaling pathway (Soleimani et al., 2020). Regarding these oncogenic signaling pathways, ceMs of UCEC, adrenocortical carcinoma (ACC), and COAD function in the p53 signaling pathway, ceMs of UCEC, and esophageal carcinoma (ESCA) function in the Ras-MAPK and mTOR signaling pathways, and ceMs of brain lower grade glioma (LGG), lymphoid neoplasm diffuse large B-cell lymphoma (DLBC), LUSC, COAD, and UCEC function in the PI3K-AKT signaling pathway (Figure 5D and Supplementary Figure 6). These findings suggest that pan-cancer ceMs regulate oncogenic signaling pathways in a flexible manner with certain similarities. The ceMs in COAD were mainly enriched in the p53, TLR, and NF-kB oncogenic signaling pathways, which regulate cell cycle arrest, DNA repair, apoptosis, proinflammatory effects, inflammation, and survival. Furthermore, the ceMs in UCEC regulated cell cycle arrest and apoptosis in the p53 pathway, proliferation and apoptosis in the Ras-MAPK pathway, lipid biosynthesis and autophagy in the mTOR pathway, and angiogenesis and DNA repair in the PI3K-Akt pathway. These results indicate that pan-cancer, where the same oncogenic pathways are regulated through the mutations-miRNA-ceRNA mechanism, is heterogeneous in specific functions. The MDM2 and MDM4 ceMs in COAD and UCEC were found to be important for the stabilization and activation of p53 and could serve as important targets for anti-cancer therapy (Toledo and Wahl, 2007; Wade et al., 2010). The NF-κB signaling pathway is a typical proinflammatory signal transduction pathway and an important target for novel anti-carcinogenic drugs (Lawrence, 2009). mTOR is critical in the pathway and promotes cancer proliferation and metabolism upon overactivation, which is also an important target for cancer therapy (Tian et al., 2019). Taken together, these results suggest that functional variations in pan-cancer that are disturbed by somatic mutations through the ceRNA mechanism may lead to tumor-specific phenotypes.

#### Survival Analysis Reveals Biomarker lncRNA in Pan-Cancer

**FIGURE 6:**
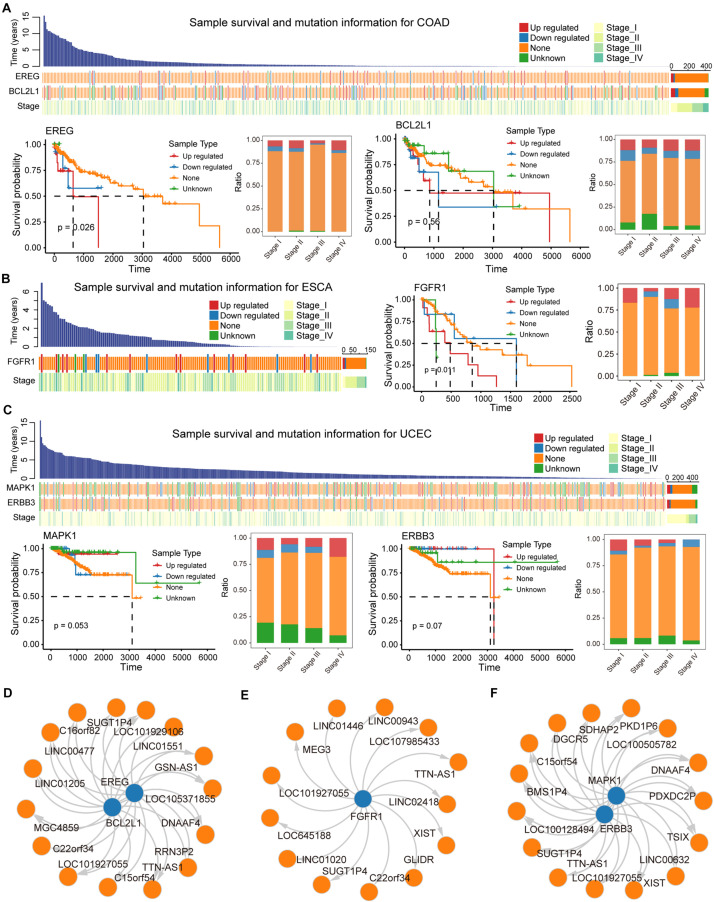
Biomarker lncRNAs (ceL) regulate mRNA (ceM) through the ceRNA mechanism. **(A)** Waterfall plot illustrating effects (Up-regulated, Down-regulate, None, and Unknown) of mutations in each sample for COAD on ceM expression, and include information such as patient survival time and clinical stage of each sample. Survival predictions and relationships to clinical staging for four sample types classified, respectively, based on the genes EREG and BCL2L1 were presented by survival curves and bar plot. **(B)** Same as in **(A)** but for ESCA, and the gene FGFR1. **(C)** The same as in **(A)** but for UCEC, and the genes MAPK1 and ERBB3. **(D–F)** The relationship between biomarker lncRNAs and regulated ceM for COAD, ESCA, and UCEC.

We evaluated the impact of ceMs that participate in the carcinogenic signaling pathway on the survival of cancer patients. Several genes (ceMs) in five cancer types had a significant hazard ratio (HR) value according to Cox proportional hazard model, suggesting a relationship between patient prognosis and lncRNA mutations (Supplementary Figure 7). The Kaplan–Meier method was used to plot survival-related ceMs (Figures 6A–C and Supplementary Figures 8A,B). We found that samples resulting in the upregulation of EREG in COAD had poor overall survival and that such samples were enriched in stage IV (Figure 6A). It is intriguing to note that according to Kaplan–Meier analysis, there was no significant difference in the survival of BCL2L1 between mutant and control samples (Figure 6A), which may be attributed to the weak effect of mutations on ceM expression in several samples. FGFR1, a proven independent prognostic risk factor in patients with resected esophageal squamous cell carcinoma (Wang Y. et al., 2019), was significantly differentially expressed in mutant ESCA samples (*p*-value = 7.56e-04), and high expression of FGFR1 was associated with poorer patient prognosis (Figure 6B). We found that “unknown” samples that perturb MAPK1 expression in UCEC had a better prognosis and the least proportion was in stage IV (Figure 6C). Previous studies have suggested that MAPK1 regulates the metastasis and invasion of cervical cancer through a ceRNA mechanism (Li W. et al., 2017). These results indicate an important contribution of the lncRNA mutation-ceRNA mechanism to the overall survival of cancer patients.

LncRNAs that regulate prognosis-related ceM expression can be critical factors in cancers. High-frequency mutated lncRNAs regulating prognosis-related ceM expression were screened for the identification of cancer-related biomarkers (Supplementary Table 3, Supplementary Figures 8C,D, and Figures 6D–F). TTN-AS1 (ENSG00000237298) was identified as a potential biomarker involved in the regulation of both EREG and BCL2L1 (Figure 6D). TTN-AS1 has been proven to be associated with the prognosis of COAD and regulate apoptosis and invasion in osteosarcoma (Fu et al., 2019), lung adenocarcinoma (Jia et al., 2019) and colorectal cancer (Wang Y. et al., 2020) via the ceRNA mechanism. The lncRNA GSN-AS1 (ENSG00000235865) regulates BCL2L1 (Figures 6D,E), which has been proven to be an important prognostic marker for luminal subtype breast cancer (Yang et al., 2016). lncRNA XIST (ENSG00000229807), TTN-AS1 (ENSG00000237298), and TSIX (ENSG00000270641), which regulate MAPK1 and ERBB3 in UCEC (Figure 6F), were shown to be miRNA sponges that control apoptosis via the ceRNA mechanism (Bu et al., 2018; Fu et al., 2019; Li et al., 2020). Taken together, these results suggest that several potential biomarker lncRNAs regulate the expression of protein-coding genes through the ceRNA mechanism to affect patient survival.

## Discussion

In this study, we integrated mutation data from 33 cancer types with RNA-seq profiles from TCGA to explore the association between somatic mutations in lncRNA and the regulatory mechanism of ceRNA. Using multivariate multiple regression and statistical analyses, we identified 162 significant HF-SMILM units and many ceMs perturbed by LF mutations from pan-cancers. The mutations-miRNA-ceRNA mechanism appeared to be dynamic, with individual differences in the regulation of ceRNA expression due to mutation specificity. In addition, we characterized the function of ceMs in pan-cancer through oncogenic signaling pathway studies and survival analysis, identifying biomarker lncRNAs that regulate the expression of ceM associated with patient survival. These findings provide a new perspective to explain the role of lncRNA mutations in post-transcriptional gene regulation.

Although we used both TargetScan and miRanda tools to predict potential SMILM units with rigorous screening of scores and energetics, it is also possible that our lncRNA mutation-miRNA detection missed target sites not predicted by either tool. Alternatively, we considered the union or intersection of multiple miRNA-target prediction algorithms, including PITA (Kertesz et al., 2007) and RNAhybrid (Kruger and Rehmsmeier, 2006); however, unions might introduce many false positives and intersections might introduce false negatives. Our current standards provide a reasonable and reliable reference for further functional research, and experimental methods such as CLIP-seq and RIP-seq can be used to overcome these shortcomings. Another potential limitation of our study is that we only considered one competing unit for detection in a complex ceRNA regulatory network. Changes in the expression of a node gene in the ceRNA regulatory network perturbs the balance of the entire network (Levine et al., 2007). The cascade effect caused by miRNA redistribution and ceRNA competition from a global perspective requires building a more complex algorithm to more accurately describe the complete response of the entire network.

It is certainly a significant idea that we consider lncRNA somatic mutations in terms of the ceRNA regulatory mechanism. The effects of genetic variation on ceRNA regulation have been revealed by previous studies (Cheng et al., 2015; Ghanbari et al., 2015; Li M. J. et al., 2017), and a large database of correlations has emerged (Li et al., 2014). However, few studies have addressed somatic mutations (Wang P. et al., 2020). Previous studies have focused on the perturbation of ceRNA mechanisms by genetic variations (Gamazon et al., 2012; Ghanbari et al., 2015; Li M. J. et al., 2017; Wang P. et al., 2020). For example, one of our prior studies determined the effect of somatic mutations of lncRNA on ceRNA mechanisms in pan-cancers (Wang P. et al., 2020). Li et al. have explored the genetic associations with ceRNA regulation in the human genome. These studies focused on methodology development and dataset construction on how to connect genomic variations and ceRNA expression. Thus, further studies evaluating the effects of mutations on ceRNA expression and downstream function are needed. Our research aimed to provide new insights into the oncogenic mechanism from the perspective of somatic mutations perturbing the ceRNA mechanism. Further, we expanded the scope of our analysis to study the effect of mutations on perturbing biological networks, functions, and clinical phenotypes. We believe that our analysis will be helpful for dissecting disease pathology caused by personalized mutations and further contribute to precision medicine.

Despite the limitations of our study, our findings reveal one of the underlying causes of changes in the physiological functions in cancer, which will help advance the development of precision medicine. Using mutation and transcriptome data from multiple cancers, we found many cancer-specific SMILM units and identified ceMs affected by ceLs. These results complement recent studies on the mechanism by which lncRNA mutations perturb ceRNA (Bhattacharya and Cui, 2016; Wang P. et al., 2020). By verifying the HF-mutation SMILM unit, we discovered the mutation-mediated ceRNA expression fluctuation mechanism. We also found individual differences in changes in the expression of ceM. The diverse distribution of HF and LF mutations in different cancers and genes was consistent with the genetic heterogeneity of different cancers. Thus, we performed a specific investigation on specific cancer types and genes based on the background of tumor heterogeneity. This strategy has been previously used to characterize individual disease pathologies caused by cancer-specific or gene-specific mutations (Lawrence et al., 2013; Zack et al., 2013; ICGC/TCGA Pan-Cancer Analysis of Whole Genomes Consortium, 2020). We believe that our exhaustive analysis will be helpful for dissecting disease pathology caused by personalized mutations and contribute to precision medicine. Importantly, we performed functional enrichment and oncogenic signature pathway studies, as well as survival analysis of ceMs from multiple cancers. We identified that pan-cancer has functional heterogeneity in mutation-ceRNA mechanisms, primarily enriched in cell proliferation and apoptosis, DNA repair, and immune regulation. Furthermore, FLT1 of LUSC, ITGB1 of DLBC, MDM4, and CDKN1A of ACC, MAPK1, and ERBB3 of UCEC, FGFR1 of ESCA, and EREG and BCL2L1 of COAD have been strongly associated with patient survival in their respective cancers. Furthermore, the biomarker lncRNAs, which regulate the above genes and are mutated with HF, contribute to the development of clinical research.

With the rapid development of high-throughput technologies, an increasing number of large biological data sets can be obtained at the whole-transcriptome level. This makes it difficult to dissect the individual pathologies behind the fast-growing datasets. Although many novel biomarkers have been identified by *in vivo* or *in vitro* experimental methods, identifying new disease-biomarker associations based on traditional, one-by-one experimental studies are expensive, complex, and time-consuming. To overcome these problems, a bioinformatics strategy has been used in previous studies to dissect gene regulation and revealed valuable results (Du et al., 2013; Li et al., 2015; Wang P. et al., 2015). We believe that our analyses will provide novel insights into mutations affecting lncRNA-associated regulatory mechanisms at the transcriptional level. Both the method and predictions could serve as helpful references for future experimental and functional dissections of lncRNAs.

## Conclusion

Our study provides a global landscape of the effects of lncRNA somatic mutations on the ceRNA mechanism in pan-cancer. Our findings extend existing knowledge on the relevance of lncRNA mutations in functions related to cancer via the ceRNA mechanism. The integration of mutation and RNA expression data from tumor samples enhances the interpretation of the identified SMILMs, helping to improve the reliability of the predictions; thus, this approach may provide more precise theoretical guidance for experimental studies and clinical applications.

## Data Availability Statement

The datasets presented in this study can be found in online repositories. The names of the repository/repositories and accession number(s) can be found in the article/Supplementary Material.

## Author Contributions

PW, BC, and YaZ conceived and designed the experiments. YuZ, PH, QG, and YH analyzed data. YQ and MX collected the data. YuZ, PH, and QG validated the method and data. YuZ and PH wrote this manuscript. All authors read and approved the final manuscript.

## Conflict of Interest

The authors declare that the research was conducted in the absence of any commercial or financial relationships that could be construed as a potential conflict of interest.

## Footnotes

1 https://portal.gdc.cancer.gov/

2 https://www.gencodegenes.org/

3 http://www.mirbase.org/ftp.shtml

4 http://mirtarbase.cuhk.edu.cn/php/index.php

5 http://software.broadinstitute.org/gsea/msigdb
