# Supplementary material for: Oncogenic Landscape of Somatic Mutations Perturbing Pan-Cancer lncRNA-ceRNA Regulation
Source: Front Cell Dev Biol. 2021 May 17;9:658346. doi: 10.3389/fcell.2021.658346 (PMC8166229; doi:10.3389/fcell.2021.658346)
Supplement: Supplementary file 1 [file Data_Sheet_1.docx]

**Supplementary Methods**

**Identification of miRNA-ceL regulation units**

The recognition of miRNA-ceL regulation can clearly recognize the influence of lncRNA somatic mutations on physiological mechanisms. First, we downloaded the lncRNA reference profile from GENCODE[[1](#_ENREF_1)] (v29, GRCH38) and mature miRNA sequences from miRBase[[2](#_ENREF_2)]. In this study, the lncRNA mutation site was used as the driver starting point. Reference and mutation sequence files for 33 cancers as well as mature miRNA sequences and annotation information, need to be prepared in advance. We used different approaches to identify miRNA-target relationships and assess the impact of genomic changes on miRNA binding sites. We used two miRNA-lncRNA target prediction methods to predict miRNA-lncRNA interactions: miRanda (v2010) and TargetScan (v.6.0), and set stringent thresholds of score > 160 and energy < -20 for miRanda[[3](#_ENREF_3)] and context score < -0.4 for TargetScan[[4](#_ENREF_4)]. Inconsistent regulatory relationships were evident between the mutant and the control sequences. Thus, a functional variant could be identified if the variant's different genotypes can alter miRNA-lncRNA interactions. We defined lncRNAs disturbed by mutations as ceL and formed miRNA-ceL regulation.

**Identification of biomarker lncRNA**

Mutations in lncNRA disturb the balance of the entire ceNRA network[[5](#_ENREF_5)]. Mutations occurring on lncRNAs are not random, and there is a specific preference for some lncRNAs[[6](#_ENREF_6)]. The higher the mutation frequency of lncRNAs, the greater the effect on variation in individual phenotypes. Target gene mRNAs (ceMs) associated with cancer patient survival were regulated by multiple lncRNAs via a mutations-miRNA-ceRNA mechanism. Of these lncRNAs affecting the same ceM, lncRNA with a higher mutation frequency are more likely to be biomarkers in the ceRNA mechanism.

**Supplementary Figures**

**
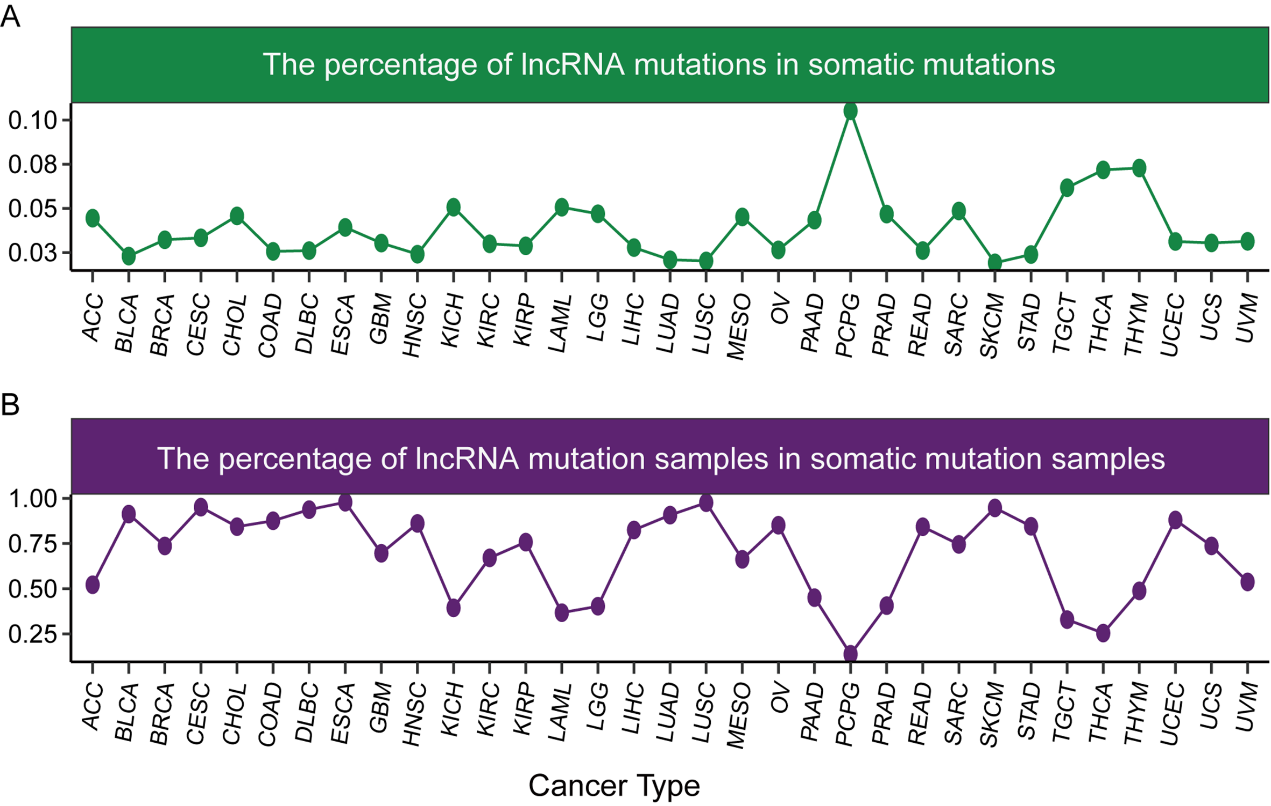
**

**Figure S1 (Related to Figure 1).** Mutation and sample data across the 33 cancer types. (A) The percentage of lncRNA mutations in somatic mutation across pan-cancer. (B) The percentage of lncRNA mutation samples in somatic mutation samples across pan-cancer.


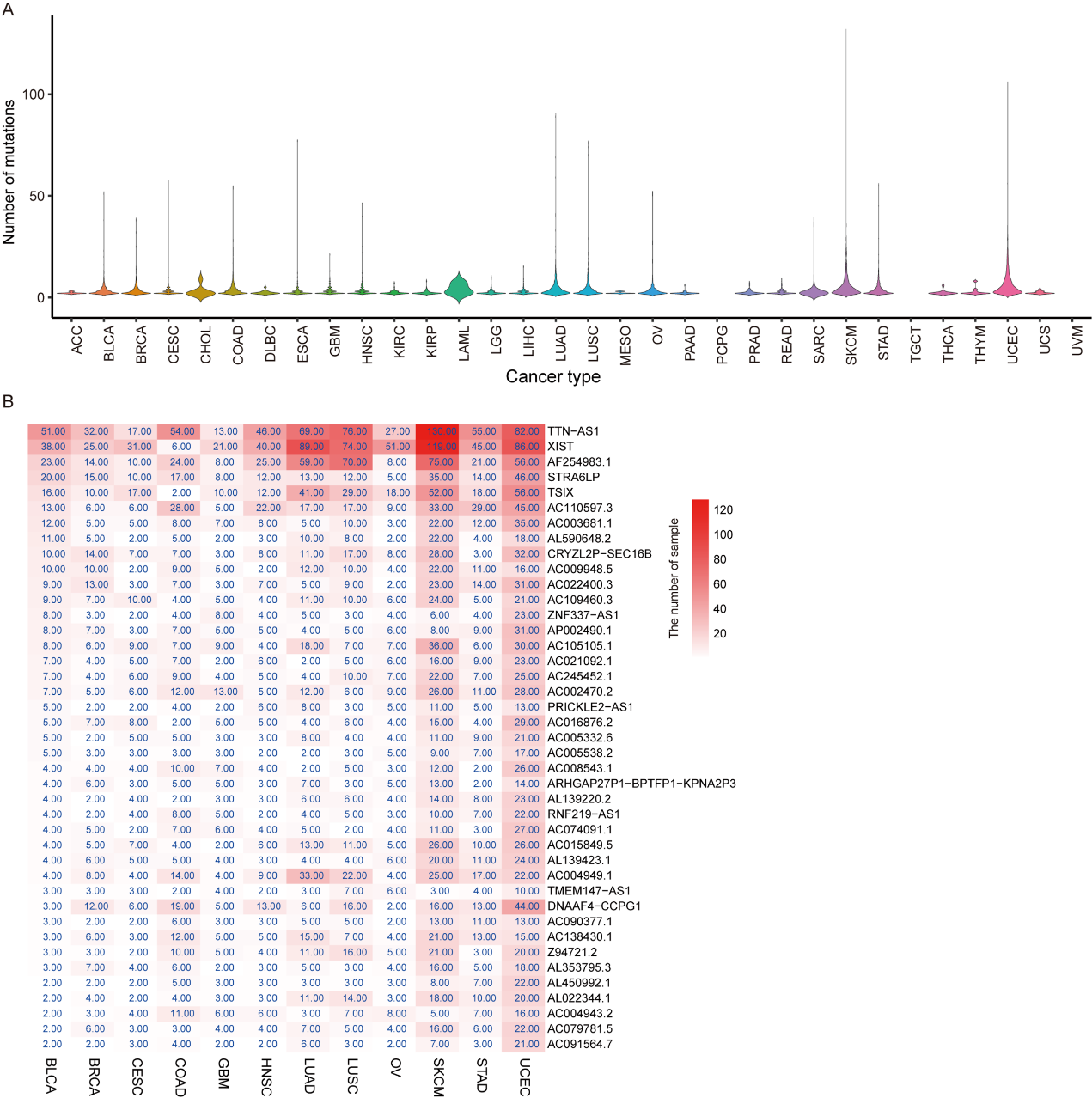


**Figure S2.** Statistics of lncRNA mutation frequency. (A) The violin chart shows the mutation frequency of lncRNA in pan-cancer, which has at least two mutations. (B) In the heat map, the horizontal and vertical coordinates show 11 cancer types and lncRNAs with high-frequency mutations and regulating prognosis-related ceMs, respectively.


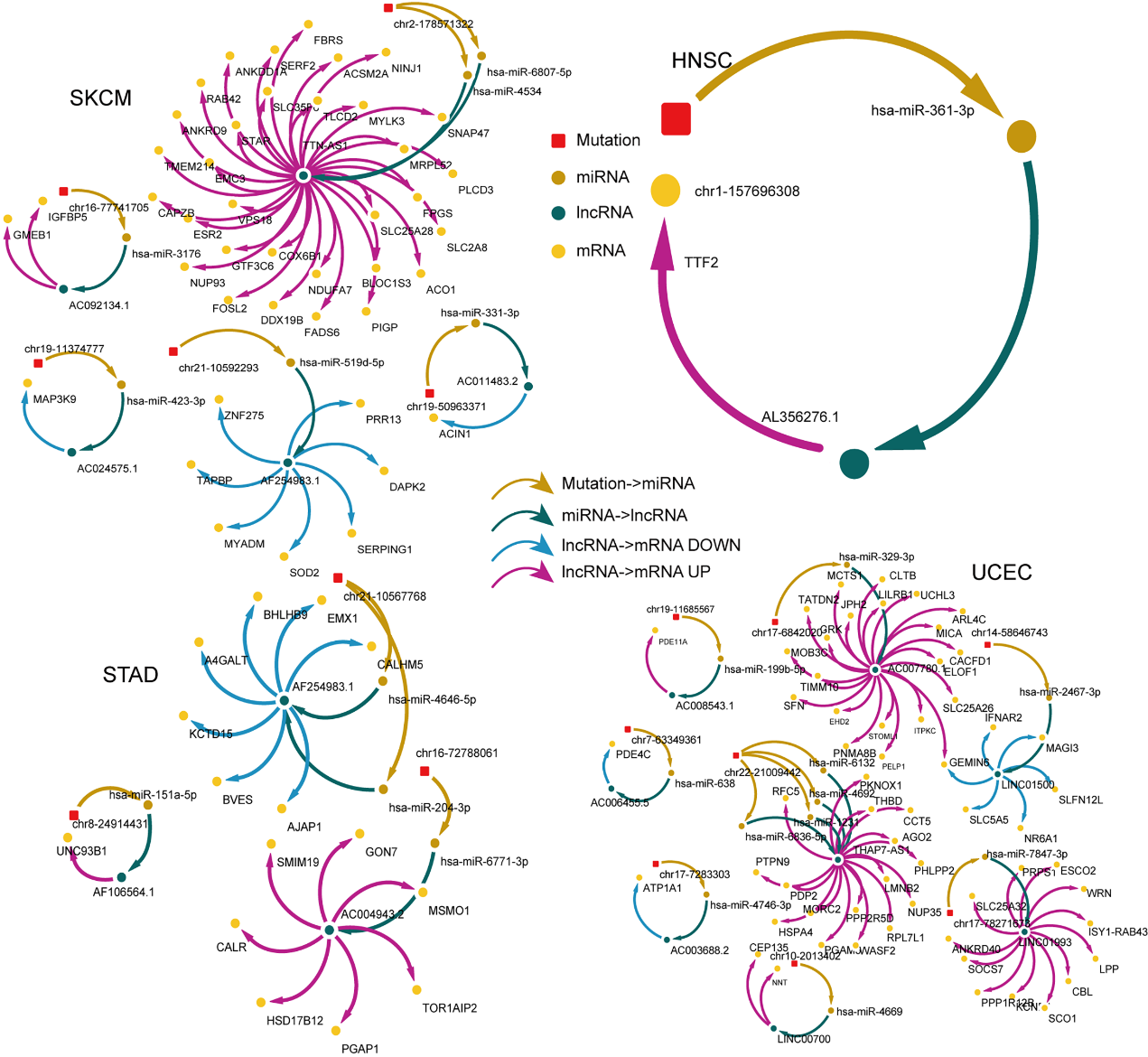


**Figure S3. (Related to Figure 3)** The ceRNA expression fluctuation landscape. (B) The same as in Figure 3D, and E but for SKCM, HNSC, STAD, and UCEC.


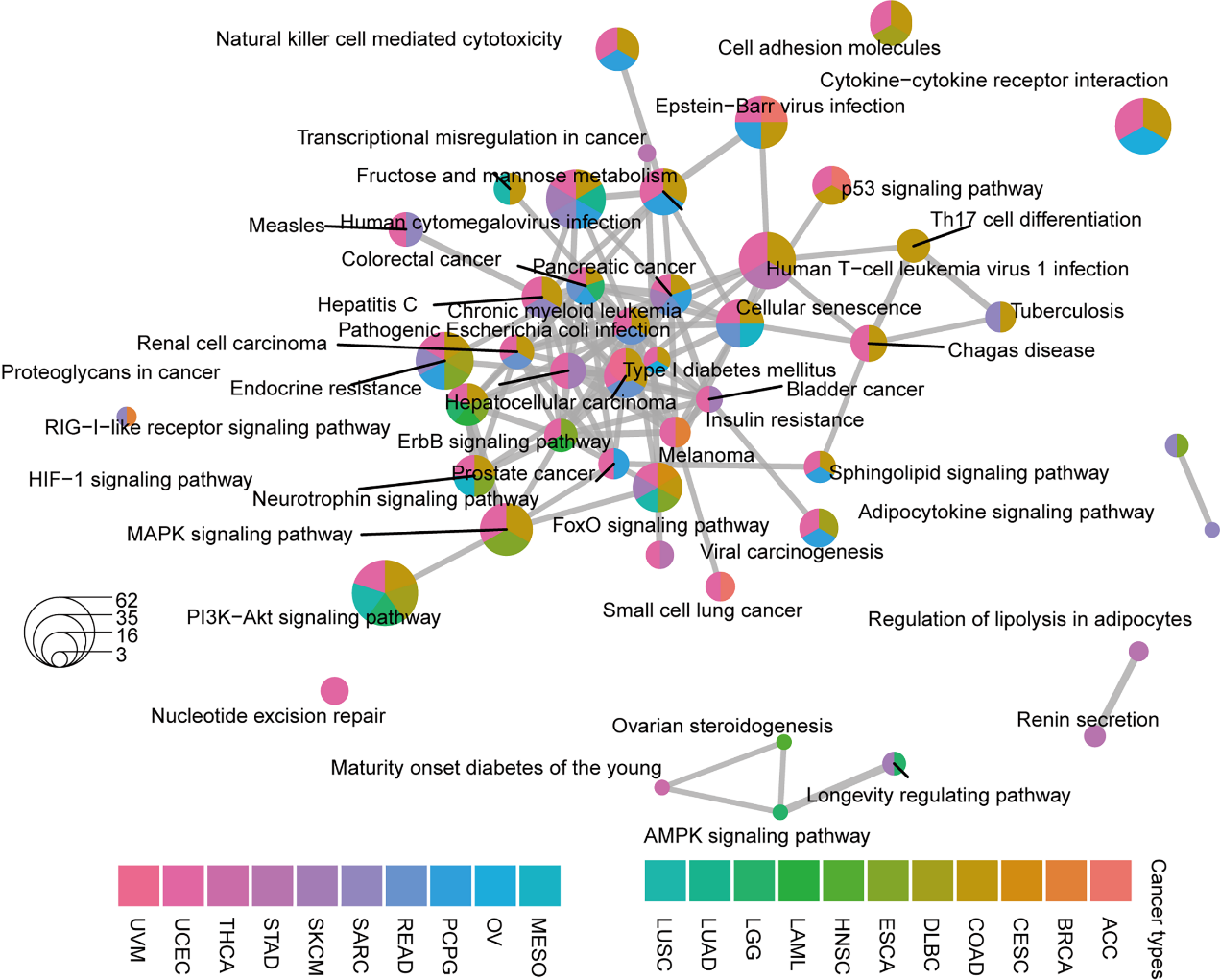


**Figure S4.** KEGG function enrichment of ceMs in pan-cancer. The network demonstrates the top five pathways in the enrichment results for pan-cancer functions.


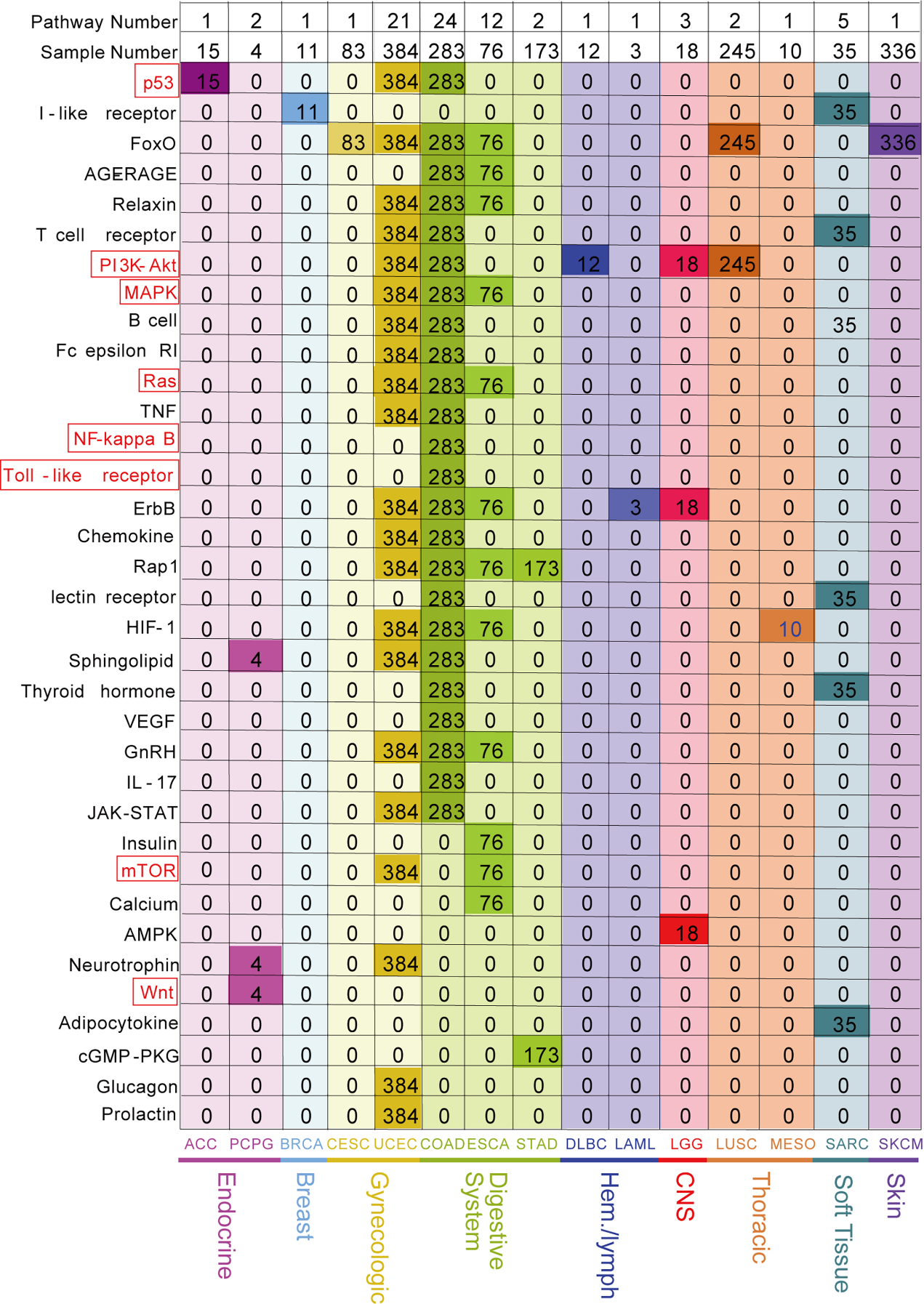


**Figure S5.** Counting samples of mutations that affect carcinogenic signaling pathways in pan-cancer. The number of all samples and pathways per cancer type were also provided.


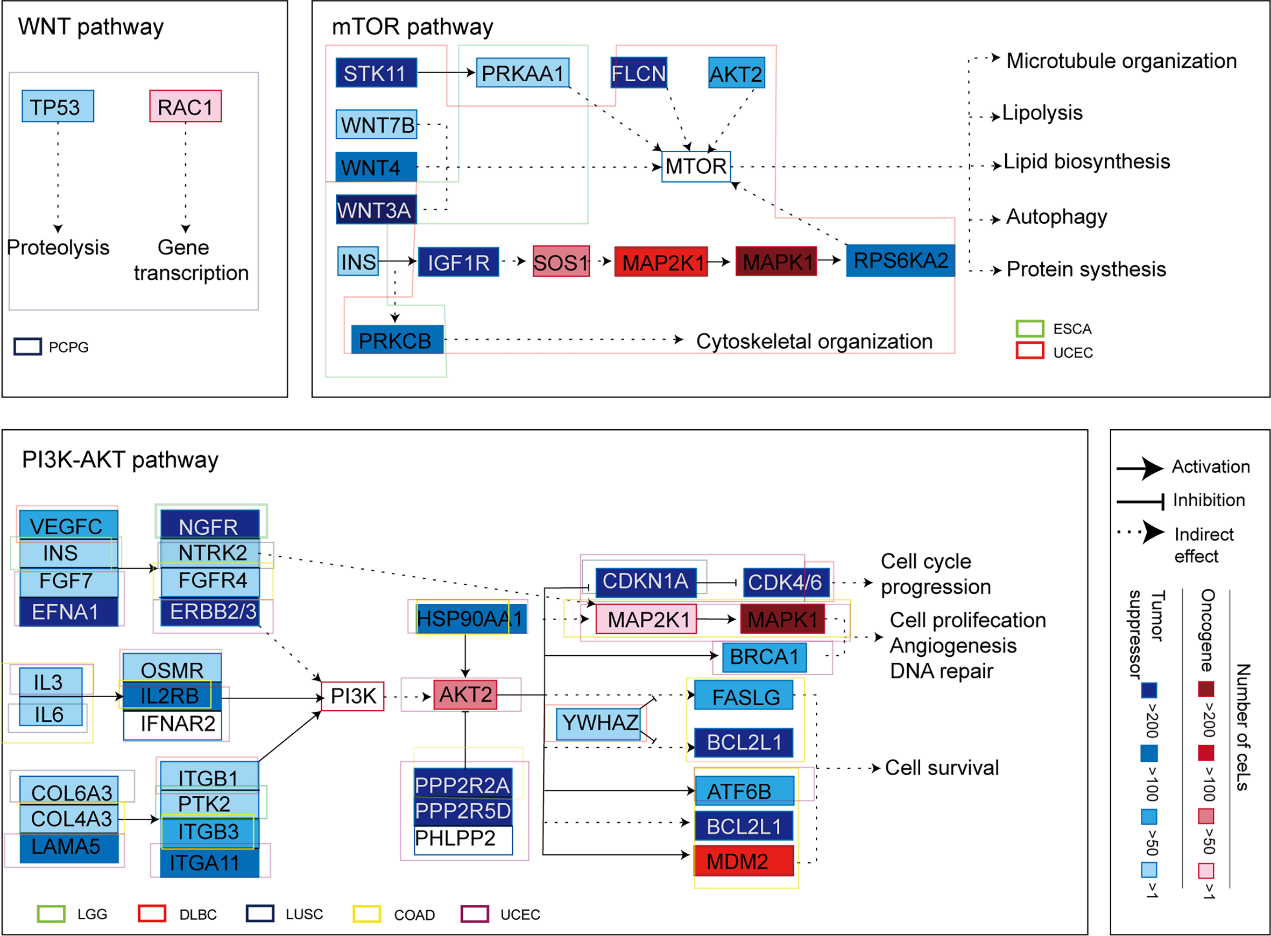


**Figure S6.** Signaling pathways affected by ceM mapped to illustrate the function mechanism of ceM in pan-cancer. Oncogenic and suppressor genes are shown in red and blue, respectively. Use of three categories to represent the relationships between two genes: activation, inhibition, and indirect effect.


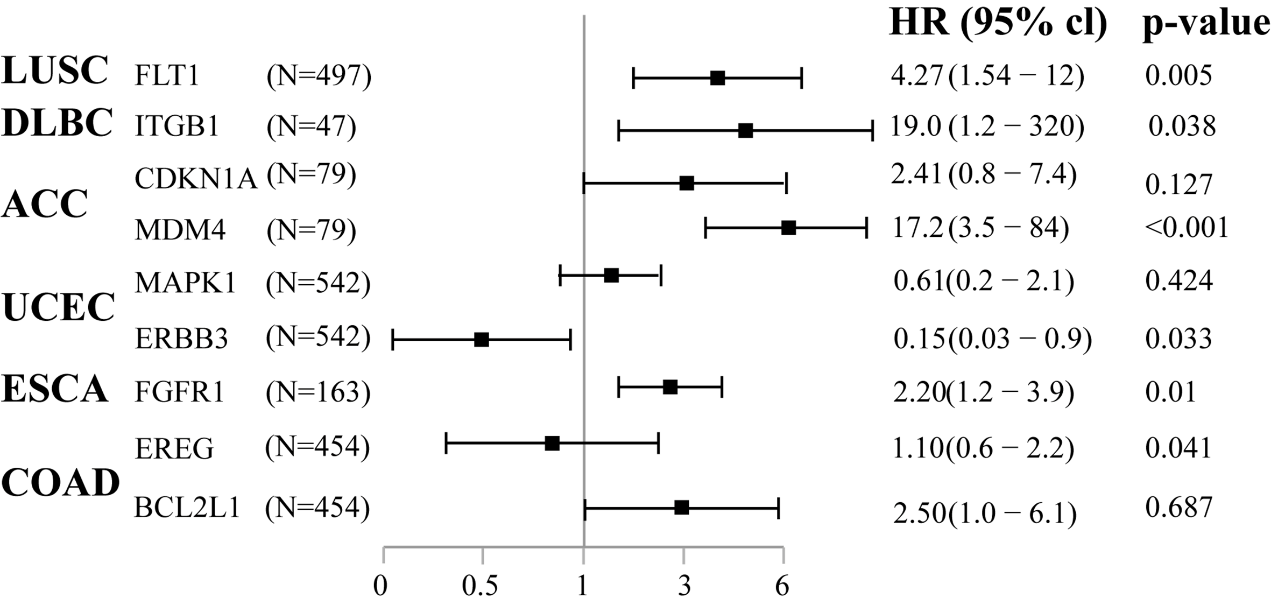


**Figure S7.** Forest plot showing results of a proportional hazards model analysis of ceMs in six cancers.

**
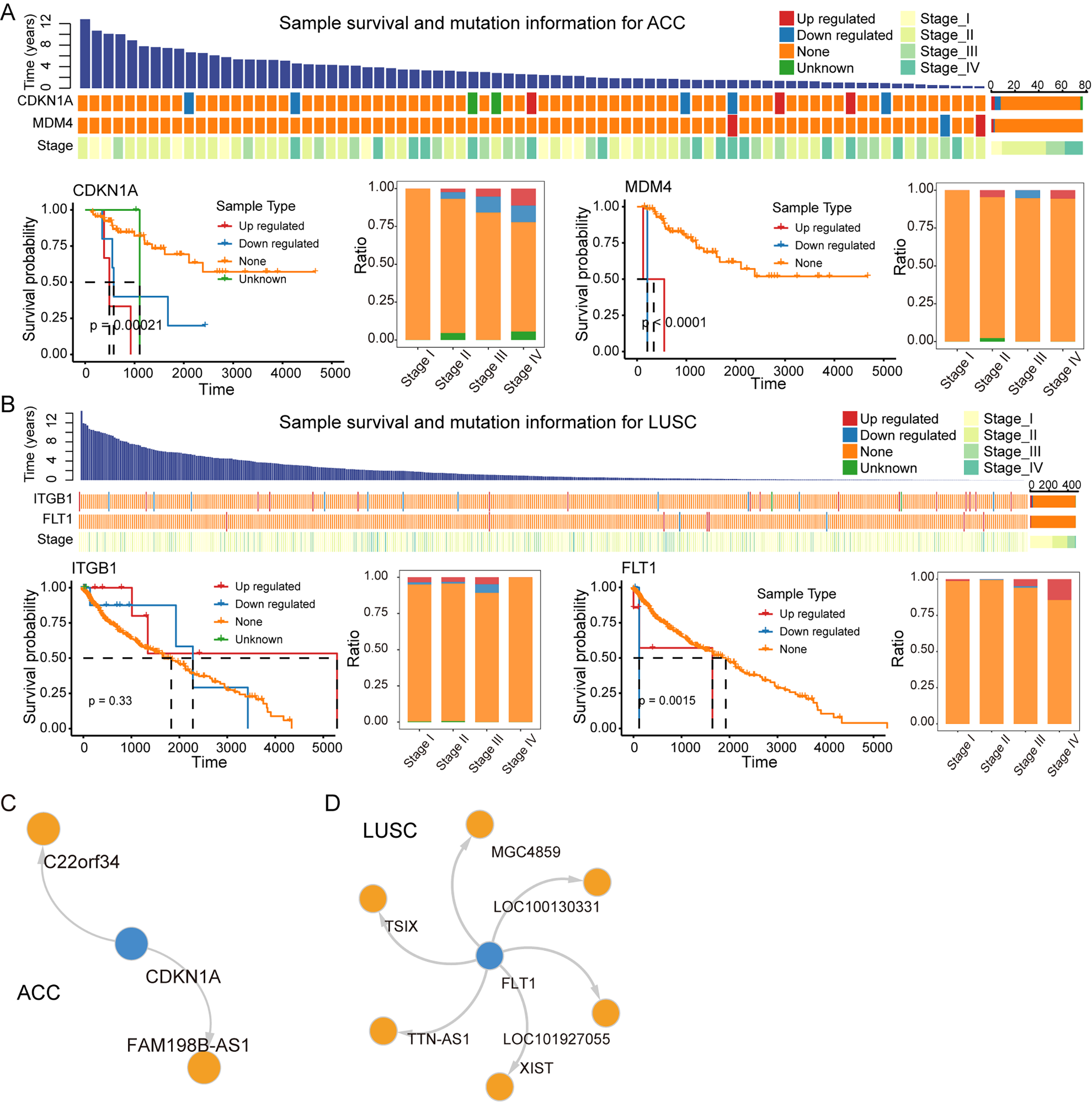
**

**Figure S8 (Related to Figure 6).** Survival-related ceM and biomarker lncRNA. (A) Waterfall plot illustrating effects (Up-regulated, Down-regulate, None, and Unknown) of mutations in each sample for ACC on ceM expression, and include information such as patient survival time and clinical stage of each sample. Survival predictions and relationships to clinical staging for four sample types classified, respectively, based on the genes CDKN1A and MDM4 were presented by survival curves and bar plot. (B) Same as in (A) but for LUSC, and the gene ITGB1 and FLT1. (C-D) The relationship between biomarker lncRNAs and regulated ceM for ACC and LUSC, respectively.

**Supplementary Tables**

| **Table S1：Samples and mutation information for 33 cancer types**   \| **Abbreviation** \| \| **Description** \| **Collected from TCGA** \| \| \| \| \| --- \| --- \| --- \| --- \| --- \| --- \| --- \| \| **Samples** \| **All_ mutation** \| **lncRNA_ mutation** \| **Proportion** \| \| ACC \| Adrenocortical carcinoma \| \| 92 \| 8408 \| 195 \| 2.32% \| \| BLCA \| Bladder Urothelial Carcinoma \| \| 413 \| 118833 \| 2493 \| 2.10% \| \| BRCA \| Breast invasive carcinoma \| \| 1051 \| 107214 \| 2544 \| 2.37% \| \| CESC \| Cervical squamous cell carcinoma and endocervical adenocarcinoma \| \| 307 \| 74045 \| 2346 \| 3.17% \| \| CHOL \| Cholangiocarcinoma \| \| 51 \| 3865 \| 149 \| 3.86% \| \| COAD \| Colon adenocarcinoma \| \| 435 \| 179930 \| 4042 \| 2.25% \| \| DLBC \| Lymphoid Neoplasm Diffuse Large B-cell Lymphoma \| \| 48 \| 6624 \| 162 \| 2.45% \| \| ESCA \| Esophageal carcinoma \| \| 185 \| 31039 \| 1191 \| 3.84% \| \| GBM \| Glioblastoma multiforme \| \| 408 \| 72076 \| 1523 \| 2.11% \| \| HNSC \| Head and Neck squamous cell carcinoma \| \| 511 \| 92881 \| 1924 \| 2.07% \| \| KICH \| Kidney Chromophobe \| \| 66 \| 1854 \| 37 \| 2.00% \| \| KIRC \| Kidney renal clear cell carcinoma \| \| 340 \| 23826 \| 478 \| 2.01% \| \| KIRP \| Kidney renal papillary cell carcinoma \| \| 289 \| 26144 \| 570 \| 2.18% \| \| LAML \| Acute Myeloid Leukemia \| \| 147 \| 6504 \| 121 \| 1.86% \| \| LGG \| Brain Lower Grade Glioma \| \| 526 \| 29826 \| 564 \| 1.89% \| \| LIHC \| Liver hepatocellular carcinoma \| \| 377 \| 44836 \| 1028 \| 2.29% \| \| LUAD \| Lung adenocarcinoma \| \| 571 \| 206897 \| 3920 \| 1.89% \| \| LUSC \| Lung squamous cell carcinoma \| \| 494 \| 172772 \| 3414 \| 1.98% \| \| MESO \| Mesothelioma \| \| 83 \| 3309 \| 99 \| 2.99% \| \| OV \| Ovarian serous cystadenocarcinoma \| \| 443 \| 69803 \| 1573 \| 2.25% \| \| PAAD \| Pancreatic adenocarcinoma \| \| 182 \| 25452 \| 497 \| 1.95% \| \| PCPG \| Pheochromocytoma and Paraganglioma \| \| 182 \| 2076 \| 30 \| 1.45% \| \| PRAD \| Prostate adenocarcinoma \| \| 497 \| 21787 \| 414 \| 1.90% \| \| READ \| Rectum adenocarcinoma \| \| 159 \| 47472 \| 1045 \| 2.20% \| \| SARC \| Sarcoma \| \| 259 \| 20837 \| 753 \| 3.61% \| \| SKCM \| Skin Cutaneous Melanoma \| \| 472 \| 404943 \| 7342 \| 1.81% \| \| STAD \| Stomach adenocarcinoma \| \| 440 \| 151982 \| 3069 \| 2.02% \| \| TGCT \| Testicular Germ Cell Tumors \| \| 155 \| 2709 \| 55 \| 2.03% \| \| THCA \| Thyroid carcinoma \| \| 503 \| 18069 \| 330 \| 1.83% \| \| THYM \| Thymoma \| \| 123 \| 2814 \| 100 \| 3.55% \| \| UCEC \| Uterine Corpus Endometrial Carcinoma \| \| 543 \| 581356 \| 15996 \| 2.75% \| \| UCS \| Uterine Carcinosarcoma \| \| 57 \| 7940 \| 178 \| 2.24% \| \| UVM \| Uveal Melanoma \| \| 80 \| 1247 \| 21 \| 1.68% \|   * Reference data used from GENCODE database |
| --- | --- | --- | --- | --- | --- | --- | --- | --- | --- | --- | --- | --- | --- | --- | --- | --- | --- | --- | --- | --- | --- | --- | --- | --- | --- | --- | --- | --- | --- | --- | --- | --- | --- | --- | --- | --- | --- | --- | --- | --- | --- | --- | --- | --- | --- | --- | --- | --- | --- | --- | --- | --- | --- | --- | --- | --- | --- | --- | --- | --- | --- | --- | --- | --- | --- | --- | --- | --- | --- | --- | --- | --- | --- | --- | --- | --- | --- | --- | --- | --- | --- | --- | --- | --- | --- | --- | --- | --- | --- | --- | --- | --- | --- | --- | --- | --- | --- | --- | --- | --- | --- | --- | --- | --- | --- | --- | --- | --- | --- | --- | --- | --- | --- | --- | --- | --- | --- | --- | --- | --- | --- | --- | --- | --- | --- | --- | --- | --- | --- | --- | --- | --- | --- | --- | --- | --- | --- | --- | --- | --- | --- | --- | --- | --- | --- | --- | --- | --- | --- | --- | --- | --- | --- | --- | --- | --- | --- | --- | --- | --- | --- | --- | --- | --- | --- | --- | --- | --- | --- | --- | --- | --- | --- | --- | --- | --- | --- | --- | --- | --- | --- | --- | --- | --- | --- | --- | --- | --- | --- | --- | --- | --- | --- | --- | --- | --- | --- | --- | --- | --- | --- | --- | --- | --- | --- | --- | --- | --- | --- | --- | --- | --- | --- | --- | --- | --- | --- | --- | --- | --- | --- | --- | --- | --- | --- | --- | --- | --- | --- | --- | --- | --- | --- | --- | --- | --- | --- | --- | --- | --- | --- | --- |

**Table S2. Overview of the 17 oncogenic pathways selected for study.**

| Pathway | Representative Genes | PMID |
| --- | --- | --- |
| Cell Cycle | CDKN2A/B, CCNDs, CDKs | 19238148 |
| HIPPO | LATS1/2, YAP1, TAZ (WWTR1) | 23467301 |
| MYC | MYC, MAX, MGA | 14663479 |
| NOTCH | NOTCHx, JAGx, EP300, CREBBP | 14570040 |
| NRF2 | NFE2L2, KEAP1, CUL3 | 22810811 |
| PI3K | PIK3CA, PTEN, AKTs, MTOR | 23863157 |
| RTK/RAS | RTKs, RAS, BRAF, MAP2K1, NF1 | 12778136 |
| TGFβ | SMADs, TGFBR1/2, ACVR2A/B | 18662538 |
| TP53 | TP53, CDKN2A, ATM, MDM2/4 | 23303139 |
| WNT | APC, CTNNB1, FZDs, RNF43 | 15829953 |
| NF-κB | TLR, TNFR, CDK-2, IL-6 | 31669643 |
| Hedgehog | PTCH1, PTCH2, GLI1 | 29274272 |
| cGAS-STING | OIS, TIS, CDK4/6 | 31799772 |
| TLR4 | TLR4, STAT3, COX-2, PGE2 | 30213077 |
| Toll like receptor (TLR) | TLRI-TLR11, IL-1, IRAK-M, IRAK | 29150944 |
| RAS / MAPK | MAP2/3K, MAPK, ERK5 | 31512778 |
| mTOR | MTOR,4E-BP1, PIP2 | 31075885 |

**Table S3.** **Biomarker lncRNAs in five cancer types.**

| Tumor type | SYMBOL (ceM) | Biomarker lncRNA (ceL) | Oncogenic Pathways |
| --- | --- | --- | --- |
| ACC | CDKN1A | ENSG00000188511, ENSG00000248429, ENSG00000125899 | P53 |
| COAD | EREG | ENSG00000237298, ENSG00000263424, ENSG00000273840, ENSG00000175746, ENSG00000278722 | MAPK，PI3K-Akt |
|  | BCL2L1 | ENSG00000237298, ENSG00000263424, ENSG00000273840, ENSG00000175746, ENSG00000278722, ENSG00000261771, ENSG00000235865, ENSG00000188511, ENSG00000255036, ENSG00000279437, ENSG00000232633, ENSG00000234665, ENSG00000228980 | Ras |
| ESCA | FGFR1 | ENSG00000279072, ENSG00000229807, ENSG00000273840, ENSG00000205611, ENSG00000237298, ENSG00000188511, ENSG00000255036, ENSG00000263424 | MAPK，Ras |
| LUSC | FLT1 | ENSG00000237298, ENSG00000229807, ENSG00000273840, ENSG00000270641, ENSG00000283117, ENSG00000237250, ENSG00000267784, ENSG00000254154 | PI3K-Akt |
| UCEC | MAPK1 | ENSG00000279072, ENSG00000229807, ENSG00000237298, ENSG00000270641, ENSG00000273840, ENSG00000281344, ENSG00000255036, ENSG00000263424, ENSG00000261771, ENSG00000231633, ENSG00000279159, ENSG00000217576, ENSG00000273419, ENSG00000242086, ENSG00000254154, ENSG00000242288, ENSG00000251143 | mTOR, Ras |
|  | ERBB3 | ENSG00000279072, ENSG00000229807, ENSG00000237298, ENSG00000270641, ENSG00000273840, ENSG00000281344, ENSG00000255036, ENSG00000263424, ENSG00000261771, ENSG00000231633, ENSG00000279159, ENSG00000217576, ENSG00000273419, ENSG00000242086, ENSG00000254154, ENSG00000242288, ENSG00000251143, ENSG00000234665, ENSG00000267257, ENSG00000279628 | PI3K-Akt |

**References**

[1] J. Harrow, A. Frankish, J.M. Gonzalez, E. Tapanari, M. Diekhans, F. Kokocinski, B.L. Aken, D. Barrell, A. Zadissa, S. Searle, I. Barnes, A. Bignell, V. Boychenko, T. Hunt, M. Kay, G. Mukherjee, J. Rajan, G. Despacio-Reyes, G. Saunders, C. Steward, R. Harte, M. Lin, C. Howald, A. Tanzer, T. Derrien, J. Chrast, N. Walters, S. Balasubramanian, B. Pei, M. Tress, J.M. Rodriguez, I. Ezkurdia, J. van Baren, M. Brent, D. Haussler, M. Kellis, A. Valencia, A. Reymond, M. Gerstein, R. Guigo, and T.J. Hubbard, GENCODE: the reference human genome annotation for The ENCODE Project. Genome Res 22 (2012) 1760-74.

[2] A. Kozomara, and S. Griffiths-Jones, miRBase: annotating high confidence microRNAs using deep sequencing data. Nucleic Acids Res 42 (2014) D68-73.

[3] D. Betel, M. Wilson, A. Gabow, D.S. Marks, and C. Sander, The microRNA.org resource: targets and expression. Nucleic Acids Res 36 (2008) D149-53.

[4] R.C. Friedman, K.K. Farh, C.B. Burge, and D.P. Bartel, Most mammalian mRNAs are conserved targets of microRNAs. Genome Res 19 (2009) 92-105.

[5] L. Wang, K.B. Cho, Y. Li, G. Tao, Z. Xie, and B. Guo, Long Noncoding RNA (lncRNA)-Mediated Competing Endogenous RNA Networks Provide Novel Potential Biomarkers and Therapeutic Targets for Colorectal Cancer. Int J Mol Sci 20 (2019).

[6] Y. Gao, X. Li, H. Zhi, Y. Zhang, P. Wang, Y. Wang, S. Shang, Y. Fang, W. Shen, S. Ning, S.X. Chen, and X. Li, Comprehensive Characterization of Somatic Mutations Impacting lncRNA Expression for Pan-Cancer. Mol Ther Nucleic Acids 18 (2019) 66-79.
